# A Data Driven Network Approach to Rank Countries Production Diversity and Food Specialization

**DOI:** 10.1371/journal.pone.0165941

**Published:** 2016-11-10

**Authors:** Chengyi Tu, Joel Carr, Samir Suweis

**Affiliations:** 1 Department of Physics and Astronomy, University of Padova, Padova, 35131, Italy; 2 Department of Environmental Sciences, University of Virginia, Charlottesville, 22904-4123, United States of America; Northwestern University, UNITED STATES

## Abstract

The easy access to large data sets has allowed for leveraging methodology in network physics and complexity science to disentangle patterns and processes directly from the data, leading to key insights in the behavior of systems. Here we use country specific food production data to study binary and weighted topological properties of the bipartite country-food production matrix. This country-food production matrix can be: 1) transformed into overlap matrices which embed information regarding shared production of products among countries, and or shared countries for individual products, 2) identify subsets of countries which produce similar commodities or subsets of commodities shared by a given country allowing for visualization of correlations in large networks, and 3) used to rank country fitness (the ability to produce a diverse array of products weighted on the type of food commodities) and food specialization (quantified on the number of countries producing a specific food product weighted on their fitness). Our results show that, on average, countries with high fitness produce both low and high specializion food commodities, whereas nations with low fitness tend to produce a small basket of diverse food products, typically comprised of low specializion food commodities.

## Introduction

By providing powerful theoretical tools and incentivizing innovative steps towards the comprehension and synthesis of broad empirical facts from increasingly available datasets, both networks physics and complexity science are actively contributing to our understanding of ecological [1, 2], environmental [3–5], economic [6–9] and social processes [10, 11]. As demonstrated by Azaele et al. [12] and many other authors [13–15], quantitative data driven approaches allow for disentangling the key drivers (e.g. birth, death, migration, adaptation and niche differentiation) of ecological emergent patterns found in several ecosystems. They show that many ubiquitous patterns can be described by a few basic ecological processes, which in turn can be incorporated into simple conceptual models. In a similar manner, patterns of human mobility which incorporate complex details of social and individual behavior can be readily described by simpler statistical models [16, 17]. As another example, it has been shown that only few economic or environmental variables are necessary to quantify the statistics of the relative fluxes of goods (measured in $) or of water (using virtual water [18]) among nations [19, 20] as well as reconstruct many properties of corresponding trade networks.

The above claim is well illustrated by a series of recent articles in which, based on economic data, the authors were able to measure the intangible “capabilities” of countries and define the economic product “complexity” [6–9, 21]. Hidalgo and Hausmann [21] have been the first to introduce the idea that both the diversity of products and a nation’s production capabilities can be inferred directly from the structure of the country-product matrix. In their view: 1) products that are ubiquitous should not require sophisticated capabilities to produce, and as such can be produced by most countries, and 2) countries with diversified production should possess many capabilities that allow for the production of sophisticated, or more complex, commodities. From this perspective, the most diversified nations are expected to be the high ranked in terms of global economic competition (or fitness). Similarly, ubiquitous products are more likely to be products with low sophistication. To quantify these concepts, Hidalgo and Hausmann [21] introduced the so-called “reflections method” through which they obtain country economic competitiveness and production complexity [21]. Unfortunately, this method suffers from both conceptual and practical problems as demonstrated in Cristelli et al. [7]. To overcome these problems, recent efforts [6, 7, 9] have defined a new method to determine the relative strength of countries and export products. This method relies on the introduction of coupled non-linear maps between country fitness and product complexity (see below for a more detailed definition) characterized by a fixed point. By exploiting the information contained in a matrix comprised of the detailed export of each country and iteratively combining row and column measures on the matrix, the authors were able to extract a relationship between the export basket of a given country and that country’s economic competitiveness and product complexity. This relationship was expected to exist based on an established economic idea known as the Ricardo hypothesis [22]. The Ricardo hypothesis suggests that: economic competitive nations should export only products comprised of a high level of sophistication (e.g. airplanes), while poor countries should export only the products which require no special capabilities (e.g. hammers). Contrary to Ricardo hypothesis, the above studies found that most developed countries export almost all products from simple (including those not requiring any level of technology) to complex, while less developed countries were able to only produce and subsequently export only relatively simple products (e.g. products which require little technological sophistication).

Inspired by these works, herein we examine the relationship between countries and the food products they produce. As there clearly exist constraints from climate, land, water and technology, on the production capabilites every country, is there an analog of the Ricardo hypothesis when considering food production? By generalizing the theoretical framework presented in Tacchella et al [9], we seek to answer the following questions: 1) Are there ubiquitous or “low specialization” food commodities that are produced by the majority of countries (the analog of the hammers)? 2) On the other hand what are the “high specialization” food products that only a select few countries are capable of producing (an analog to airplanes)?

We also seek to evaluate the analog of economic fitness for each nation in terms of food production capability. Can we identify high fitness countries that can potentially sell not only several different food products, but also food products that are also highly specialized (i.e. can they place on the market food commodities that the other nations are not able to produce)? In contrast, can low fitness countries produce only food commodities of low specialization (i.e. can they only place on the market food commodities that the other nations are able to produce)? Using the proposed theoretical framework, we set out to generate an unbiased quantification of: 1) specialization for all food products and 2) fitness for all the countries.

## Materials and Methods

### Food Production Data and Network Analysis

Food production and bilateral trade data in tons was extracted from the United Nations Food and Agricultural Organization database (FAOSTAT). In this study we only consider primary commodities (e.g. wheat) and we do not include secondary commodities (e.g. bread). Fish production data was also extracted from the FAOSTAT database and include the production data for eight primary fish commodities (freshwater fish, crustaceans, demersal fish, pelagic fish, marine fish, cephalopods, mollusks, aquatic mammals). The data was limited to the interval 1992-2011 and only countries with population greater than half million people were considered. Country separations and merges during this time period were handled following [23] resulting in a final data set that covers 157 commodities for 177 countries [24].

From this data the country-food production adjacency matrix *M*_*cp*_ (*y*) is constructed for the year *y*, where *M*_*cp*_ (*y*) = 1 if country *c* produces product *p* in year *y*, and zero otherwise. We then build the weighted country-food per capita production matrix *W*_*cp*_ where $W_{cp}\;(y)=\frac{ton_{cp}(y)}{pop_{c}(y)}$, where for each year *y*, *ton*_*cp*_ is the production volume (in tons) of the food commodity *p* produced by country *c* while, *pop*_*c*_ is the population of country *c*. To obtain country import graphs *I*_*ci*_ (*y*) we used data on the volumes of food commodities imported by each country for each primary commodity: $I_{ci}\;(y)=\frac{ton_{ci}(y)}{pop_{c}(y)}$. We note to the reader that production and import fluxes can be weighted in different ways by appropriately converting the unit of measure (e.g. we can transform tons to calories, by multiplying food commodities volumes by caloric content).

**Fig 1:**
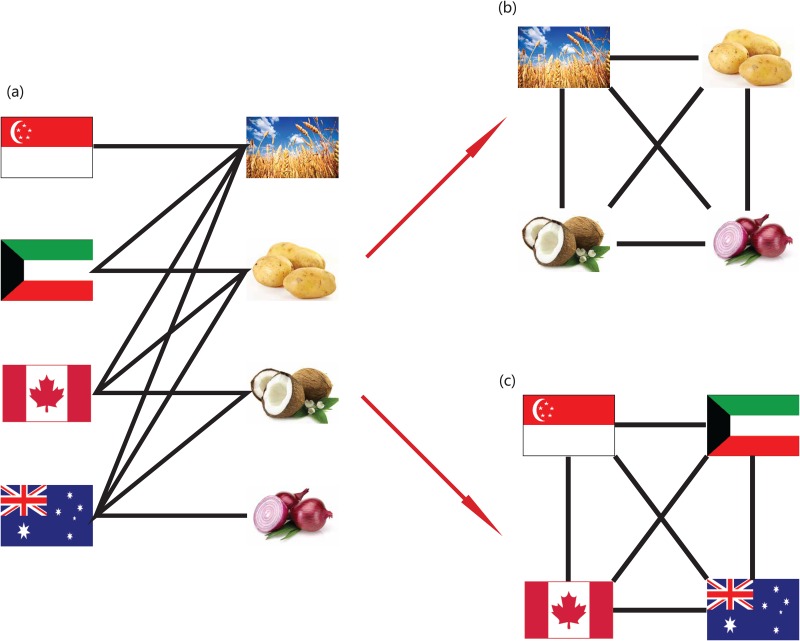
Bipartite country-food production network and its projection networks. A simple example showing how the two projection networks arise from the bipartite country-food production network (a) which describes the different food commodities produced by each country. (b) Food-food projection network obtained from the bipartite graph shown in (a). In this projection, nodes represent different food commodities which are connected to each other if they both are produced by the same country. (c) Country-country network obtained from the bipartite graph shown in (a) where nodes represent countries and countries are connected if they produce the same food commodity.

In order to obtain a first order understanding of the relationships among countries induced by their food production (and vice-versa), we can study binary and weighted topological properties of the bipartite country-food production matrix (Fig 1 (a)). This type of analysis is relatively standard [25], and thus herein we only analyze a few properties that are relevant for the main objectives of this work. Let *M*_*cp*_ be the adjacency matrix of the country-food production graph. Then the number of food commodities produced by country *c* is $k_{c}^{P}=\sum_{p}M_{cp}$ while the number of countries that produce food commodity *p* is $k_{p}^{C}=\sum_{c}M_{cp}$. Thus the number of food commodities produced both by country *i* and *j* is $o{}_{ij}^{P}=\sum_{p}M_{ip}M_{jp}$ (*o* is known as overlap matrix [26]). Analogously, the number of countries that produce both food products *u* and *w* is $o{}_{uw}^{C}=\sum_{c}M_{cu}M_{cw}$.

Nestedness [27] is a global network measure that is a function of the above overlap has been used to describe hierarchy in the interactions in ecological networks (e.g. mutualistic plant-pollinators communities [1, 2]). The nestedness based on overlap and decreasing fill (NODF) is defined as

$$NODF=\frac{\sum_{i<j:i,j\in C}T_{ij}^{C}+\sum_{i<j:i,j\in P}T_{ij}^{P}}{\left[\frac{C\left(C-1\right)}{2}\right]+\left[\frac{P\left(P-1\right)}{2}\right]},$$ (1)

where $T_{ij}^{X}=0$ if $k_{i}^{X}=k_{j}^{X}$ and TijX=oijX/oijXmin(kiX,kjX)min(kiX,kjX) when both *i* and *j* belong to the same set *X* = *C*, *P* (the number of countries and food products respectively) [28]. To understand if the country-food production graph is nested, we first calculate the NODF of *M*_*cp*_(*y*), we then build 100 random networks (where links are placed at random) with the same size and connectivity defined as the the fraction of non-zero interactions, then calculate the NODF of these graphs. We finally compare the NODF of empirical data versus the distribution of NODF values from the random network models.

### Projection Graphs and Minimum Spanning Forest Algorithm

Important information on food production patterns can be also obtained from the weighted country-food production matrix *W* by building projection graphs [25] (see Fig 1(b) and 1(c)). In projection graphs, nodes represent countries (or food commodities) and a link is placed if two countries produce the same food commodity (or if two food products are produced by the same country). This connects the various countries (or food products) with an undirected link whose strength is given by the number of products mutually produced (or the number of countries producing the same given food). This method allows for the information stored in the matrix *W* to be projected into networks of country-country and food-food products as shown in Fig 1. The country-country network is characterized by the *N*_*C*_ × *N*_*C*_ country-country matrix *C* = *WW*^*T*^. The non-diagonal elements *C*_*cc*′_ correspond to the number of products that countries *c* and *c*′ have in common. The diagonal elements *C*_*cc*_ corresponds to the number of products produced by country c and are a measure of the diversification of country c.

We can use projection graphs to quantify the correlation in the production among two countries. We first define the similarity matrix among countries as $S_{cc^{\prime }}=\frac{2C_{cc^{\prime }}}{C_{cc}+C_{c^{\prime }c^{\prime }}}$, where 0 ≤ *S*_*cc*′_ ≤ 1 and the larger the value, the stronger is the correlation between the food commodities produced by the two countries *c* and *c*′. From the similarity matrix *S*, we find the Minimal Spanning Forest (MSF) of the projection networks (with the constraint that no edge between two nodes is allowed if they have already been connected to some other node) in order to visualize only the strongest correlations among countries or food products [6]. This method generates a set of disconnected sub-trees (i.e. a forest) to divide the country-country projection graph into different communities characterized by strong overlaps in food production patterns. The MSF algorithm is structured by performing the following steps:

1. Sort all the edges of the similarity matrix *S* by their weight (from the largest to smallest weight);
2. Add one edge at time from the sorted vector obtained in Step 1. If the two nodes of the new edge have been previously connected via an edge, do not add this edge.
3. Repeat Step 2 until all the potential edges are added.

In this manner, the MSF naturally splits a network of countries into separate subsets allowing for clear visualization of the correlations that exist in the larger network. This same analysis can be performed for the food-food network where the communities identified from the MSF algorithm represent baskets of food products which share similar producers. Similarly, we can apply the same method to a weighted country-food import matrix *I* instead of the weighted country-food production matrix *W* in order to examine the patterns in food importation.

### Calculation of the Country Fitness and Food Specialization

Here we describe the non-linear iterative procedure proposed in Tacchella et al. [9] that we apply to determine the country fitness and food specialization. This method seeks to quantify a country’s fitness *F*_*c*_, a measure of its ability to produce a diversified and specialized food basket, and to evaluate food specialization *Q*_*p*_, food products that are produced only by few high fitness countries. In mathematical terms, fitness and specialization define a new metric for determining the relative centrality of countries and products in the context of food production.

The general concept hinges on defining an iterative process which both couples, and estimates, *F*_*c*_ and *Q*_*p*_. Final estimation of *F*_*c*_, *Q*_*p*_ are then achieved in the limit of large number of iterations. This non-linear iterative process can be defined in the following way. First, the country fitness *F*_*c*_ is assumed to be proportional to the sum of the different food commodities produced in that nation, weighted by their specialization *Q*_*p*_. Second, *Q*_*p*_ is considered to be inversely proportional to the number of countries which produce it, weighted by their inverse fitness (i.e. if a country has a high fitness this should reduce the weight in calculating the food specialization, and vice versa [9]). This new estimation of *Q*_*p*_ allows for recalculation of *F*_*c*_. The non-linear relation can be seen as the fixed point equation of this iterative algorithm so that *Q*_*p*_ and *F*_*c*_ are estimated quantitatively through the attractive asymptotic fixed point. Iteration between these two calculations converge to a country fitness and the food specialization.

The following algorithm is used to determine food specialization and country fitness [9]:

1. Set the initial condition of country fitness $Q_{p}^{0}=1$ and food specialization $F_{c}^{0}=1$;
2. Compute the intermediate variables as follows $\{\begin{array}{c} {\tilde{F}}_{c}^{n}=\sum_{p}W_{cp}Q_{p}^{-1}\\ {\tilde{Q}}_{p}^{n}=\frac{1}{\sum_{c}W_{cp}\frac{1}{F_{c}^{n-1}}} \end{array}$, where *W*_*cp*_ is the element of the weighted country-food product matrix *W*, ${\tilde{F}}_{c}^{n}$ and ${\tilde{Q}}_{p}^{n}$ are the intermediate variables, *n* is the iterative step. This step can be changed to vector calculation to allow for parallel computation as follows: $\{\begin{array}{c} F^{n}=WQ^{n-1}\\ Q^{n}=\frac{1}{W^{T}\frac{1}{F^{n-1}}}. \end{array}$
3. Normalize the intermediate variables of country fitness and production specialization: $\{\begin{array}{c} F_{c}^{n}=\frac{{\tilde{F}}_{c}^{n}}{\langle {\tilde{F}}_{c}^{n}\rangle }\\ Q_{p}^{n}=\frac{{\tilde{Q}}_{p}^{n}}{\langle {\tilde{Q}}_{p}^{n}.\rangle } \end{array}$
4. Repeat the above steps until the country fitness $F_{c}^{n}$ and food specialization $Q_{p}^{n}$ have reached a fixed point solution.

**Fig 2:**
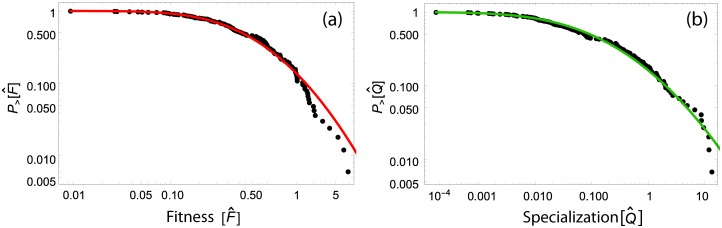
Distribution of country fitness and food specialization. The cumulative distribution of (a) country fitness and (b) production specialization. The country fitness and food specialization variables reach a fat tail distribution (Log-Normal with parameters *μ* = −0.607839, *σ* = 1.18597 and *μ* = −2.37283, *σ* = 2.40229, respectively) independently of the algorithm initial condition.

Each iteration of the algorithm adds higher order information on these quantities and a unique set of two fixed points, $F_{c}^{n}$ and $Q_{p}^{n}$, for any initial condition $F_{c}^{0}$ and $Q_{p}^{0}$ is eventually obtained. The final values of *F*_*c*_ and *Q*_*p*_ are quite heterogeneous among nations and products (i.e., after 50 iterations of the non-linear map, they evolve and their distributions follow a fat tail, Log-Normal like distribution (see Fig 2). Thus the non-linear iterative procedure leads to a unique asymptotic solutions (i.e. fixed point) and allows for the ranking of countries and food commodities based on their respective fitness and specialization.

## Results

### Country-food Production Network Properties

**Fig 3:**
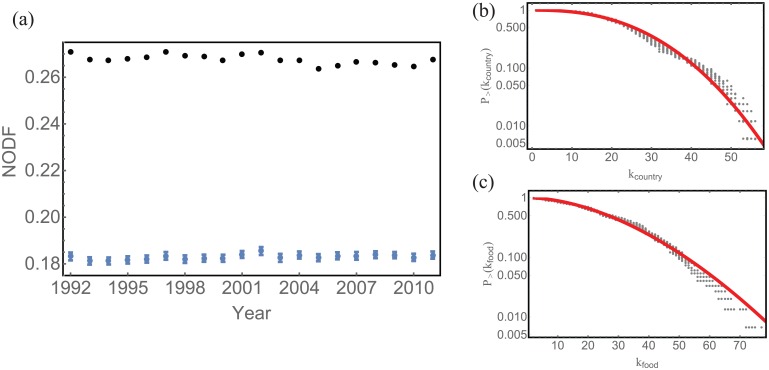
Topology of bipartite country-food product networks. (a) The nestedness (measured by the NODF given by [28] Eq (1)) of country-food production bipartite empirical networks (black point) and corresponding 100 randomly assembled networks of the same size and connectivity (blue points denote the mean and the bands are one standard deviation above and below the mean) for each year; (b) The cumulative in-degree and (c) out-degree distribution of the country-food production network for the years 1992-2011 (gray dots). These are distributed consistently with Weibull distribution (red think line) with parameters *α* = 2.510, *β* = 30.000, *μ* = −0.387 and with *α* = 1.774, *β* = 32.230, *μ* = 0.609, respectively.

For the Ricardo hypothesis to hold in our case, low fitness countries should produce low specialization food products, while nations with high fitness should only produce specialized food commodities. This would be evidenced by compartmentalized (i.e., modular), rather than nested, country-food production graphs [25]). In partial agreement with what is found for industrial and economic products [29], Fig 3(a) shows that the bipartite country-food product networks have NODF consistently higher than those found in randomly assembled networks (P-value < 0.001) for all the years analyzed (1992-2011). This result indicates that food commodities produced by countries that are only able to produce a small basket of different food products, are also produced by nations that are characterized by large and diversified food production. This nested topological structure indicates that specialized food commodities (low *k*_*food*_) are in general produced by countries with high food diversity (high *k*_*country*_), while generalist “food commodities” (low specialization and high *k*_*food*_) are also produced by “specialist” countries (low *k*_*country*_). We also found that both *k*_*food*_ and *k*_*country*_, Fig 3(b) and 3(c), are distributed consistently with a Weibull distribution. The compressed exponential decay in the tail of both *p* (*k*_*food*_) and *p* (*k*_*country*_) implies a limited heterogeneity in terms of the number of products produced by a single country, and on the number of countries producing the same food product.

### Emergent Correlations in the Food Production and Import Graphs

**Fig 4:**
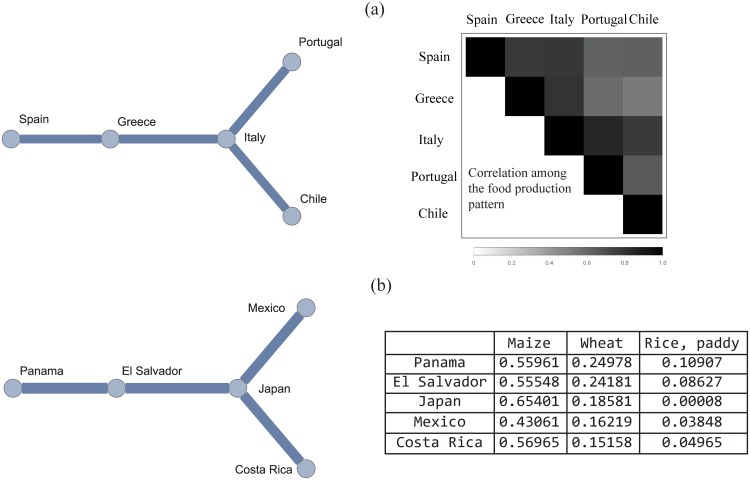
Emergent correlation in food production and import. (a) Example of a typical sub-tree from the MSF of the country-food production network for the year 2011. This sub-tree reflects the correlation in the food production among these countries, highlighted by the corresponding correlation matrix plot; (b) Example of a typical sub-tree from the MSF of country-food import network. The detected correlation in food importion among these specific countries is due to the fact that a large fraction of imports in these countries is comprised of three food commodities: maize, wheat and rice (see corresponding table).

In order to find emergent correlations in the country-food production and/or import projection graphs we apply the MSF algorithm. As explained previously, the MSF naturally splits the network of countries into distinct subsets allowing for the visualization of correlations. Small sub-trees are prevalent for all years considered in this study (1992-2011). These sub-trees possess geographical and/or climatological similarities with consequent significant correlations in food production and import patterns (see Fig 4). There are very few large sub-trees. We emphasize that, by construction, the countries comprising each forest are correlated in their production and thus potentially compete with each other in the food market. In other words, we find that there are many small groups of countries (or food products) strongly correlated with each other, and that groups with many nodes are not strongly correlated. By analyzing all MSFs, we find that developing countries primarily appear to be direct competitors of their geographical neighbors. However, edges among some countries depend on climatological similarities rather than geographical vicinity.

### Food Specialization and Country Fitness

While we can expound on the fitness and specialization of all nations, we limit our textual analysis to common socioeconomic political groupings such as the G7 (France, Germany, Italy, Japan, United Kingdom, United States of America and Canada) and BRIC (Brazil, Russian Federation, India and China).

**Fig 5:**
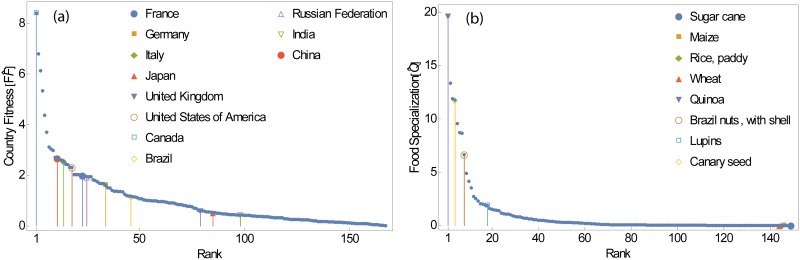
Ranks of country fitness and food specialization. (a) The rank of country fitness of countries for the year 2011 highlighting members of the G7 and BRIC countries. In general, G7 country ranks are quite high. In BRIC, the ranks of China and Russian Federation are high, Brazil is in the middle and India is quite low. (b) The rank of food specialization of food commodities for the year 2011. On one hand, the production complexity of “Sugar cane”, “Maize”, “Rice, paddy” and “Wheat” is low because their yield is very large; On the other hand, the specialization of “Quinoa”, “Brazil nuts, with shell”, “Lupins” and “Canary seed” is high because they are rare products in the market, produced only by few countries, given the particular climatic and soil condition needed to produce them.

As would be expected, country fitness of G7 countries is in general quite high (see Fig 5(a) and Table 1). However there are important differences: United Kingdom and Japan are always ranked lowest within the group, whereas the fitness of Canada is always near the top. This can be explained by UK and Japan both being island nations with territories that are not extensive, and consequently low variability in climatic regions and geography relative to other nations. This conceptually leads to a relatively low capacity to produce large quantities of diverse food commodities (in fact they have a below-average weighted degree: 0.41, 0.98 tons person^-1^ year^-1^, respectively). In contrast, Canada is large geographically, stretching from the Atlantic Ocean in the East to the Pacific Ocean in the West, and comprised of diverse climatic regions, from temperate on the West coast of British Columbia to a subarctic climate in the North. This allows for production that is various and abundant (it has a weighted degree of 2.68 tons person^-1^ year^-1^). Interestingly, within the BRIC countries, India has high variability in climate and geography, but its country fitness rank is quite low reflecting insufficient diversified food production with respect to its large population. On the other hand, China and Russian Federation like Canada both possess variable climate and geography and similarly are ranked high. For these two countries, the capacity to produce a large quantity of diverse food commodities is high. In general, if foods produced by one country are various and abundant (for example Canada, Australia and New Zealand) and/or the specialization of these foods are high (for example Bolivia and Peru), then the country fitness will be high (first column of Table 1). Vice versa, if a country has a small basket of food commonly produced also by other countries (low *k*_*food*_ and high *k*_*country*_), then the country will have a low fitness (second column of Table 1).

**Table 1:** The 10 highest and lowest fitness countries in the year 2011.

	Highest Fitness	Lowest Fitness
1	Canada	Western Sahara
2	Bolivia (Plurinational State of)	Djibouti
3	Australia	Lesotho
4	New Zealand	Singapore
5	Poland	Qatar
6	Peru	Bahrain
7	Turkey	Puerto Rico
8	Greece	Equatorial Guinea
9	Belarus	Comoros
10	Hungary	Liberia

Similarly, Fig 5(b) presents the food specialization ranking for four crops with large yield (“Sugar cane”, “Maize”, “Rice, paddy” and “Wheat”) and four products with high production specialization (“Quinoa”, “Brazil nuts, with shell”, “Lupins” and “Canary seed”). High specialization corresponds to food products that are “rare” in the market, as only few countries could produce them (low *k*_*country*_). On the other hand, food products with very large yield have low specialization (the second column of Table 2), as many countries produce these commodities. We highlight that some low specialization food commodities (e.g. wheat, potatoes, maize, and cassava) are very important in term of food security as they compose the building blocks of the diet of billions of people. Other food commodities, like sugar cane, bananas or cow milk possess large production values because they are highly requested in the food market.

**Table 2:** The 10 highest and lowest specialization food in the year 2011.

	Highest Specialization	Lowest Specialization
1	Quinoa	Sugar cane
2	Gooseberries	Cassava
3	Cranberries	Cow milk, whole, fresh
4	Canary seed	Maize
5	Blueberries	Rice, paddy
6	Bird meat, nes	Wheat
7	Meat of Asses	Potatoes
8	Brazil nuts, with shell	Bananas
9	Poppy seed	Vegetables fresh nes
10	Goose and guinea fowl meat	Plantains

## Discussion

This work applied tools and concepts from complex network science to analyze the world food production data. Based on a country-food production matrix, we analyzed the country-food production network properties and correlation among countries using MSF methodology and calculated the country fitness and food production specialization.

Our results show that there exists a hierarchical nested organization in the country-food production network. The significant nested structure observed in the country-food production graph highlights how there are many “basic” (low specialization) food products that are produced by most of the countries (the analog of “the hammers”), and few complex commodities characterized by particular climatic or geographical conditions (e.g. “the airplanes”) that are produced only by a select few countries. Thus, for country-food production, the Ricardo hypothesis does not hold as the high fitness countries produce almost all products, from the common to the specialized. Wheat, rice, maize or sugar cane are staple commodities that are the building blocks of our “food” economy whereas, Quinoa, Brazil nuts, and Lupins are produced by only a few countries (e.g. Bolivia, Peru) which in turn, although they are not the large food producers (in terms of total volumes), produce many different food products (high *k*_*country*_) and are thus also specialized in producing these “complex” commodities. The observed structure reflects the existence in a trade-off between diversification in food production, and intensification of some “optimal” commodity based on climatic-soil-water conditions.

We also found that both degree distributions and NODF values are very stable among different years (Fig 3), suggesting that the topological structure of the country-food production network has not changed significantly during the last twenty years. We leveraged a MSF algorithm in order to highlight the correlation among countries and volumes of food produced resulting in the discovery that countries with high GDP, as for example United States of America, most often belong to small sub-trees. In general, as the food production basket of these developed countries is large, it is difficult to have a high similarity with all other countries as similarity depends on the relative shared volumes. Alternatively, countries with relatively small diversity in food production, but with a pool of typical (high volume) food products tend to belong to larger sub-trees. As an example (Fig 4(a)) the second largest sub-tree is composed mainly by countries with a Mediterranean-type of climate and diet. Chile and Italy are far in term of geographical distance, but their food production is highly correlated. Similarly, one of the largest MSF production sub-trees (see SI for details) is comprised of Indonesia, Sri Lanka, Guinea-Bissau, Papua Guinea, Madagascar and Burundi: all countries that are geographically distant, but rice is the main and staple food product and the MSF algorithm detects a significant correlation structure in the production patterns of these countries (see Fig 4).

Applying the same approach to the country-food import network and building the MSF for imported food volumes (rather than for food production) provides information on the sets of countries that compete in the food market to import from the same pool of food products. Fig 4(b) shows the three largest import food commodities of the countries contained in one typical sub-tree, Panama, El Salvador, Japan, Mexico and Costa Rica with clear similarity in the food commodities that these countries import. Costa Rica and Mexico are geographically very distant from Japan, but in all these countries more than the 60% of food import is made up of maize and wheat. In general, these country-food import sub-trees may be useful to better understand indirect dependencies of a country’s food imports on certain commodities. We speculate that if there is a price spike on given food commodity shared within a sub-tree (such as maize), competition amongst members of that sub-tree whom are characterized by large import of same food commodity, will dramatically increase. This has the potential to trigger food crises in the poorest nations (those nations with limited ability to compete financially in the import market) within the corresponding sub-tree.

Our methods also provide an estimated quantification of both specialization for all food products and fitness for all the countries. We note that in our analysis, using data from 1991 to 2011, Quinoa emerges as one of the most specialized food commodity. It is interesting to note that in October 2015, imports of Quinoa to Europe increased by 40%: the same growth rate as recorded both in 2013 and in 2014 [30]. Therefore our analysis allowed us to identify specialized products with high growth potential in the food market.

**Fig 6:**
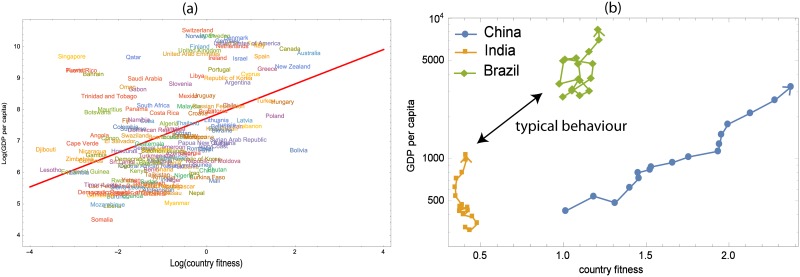
Relationship between country GDP and fitness. (a) The static country fitness—GDP per capita plane of the year 2011. We do not find significant correlation between these two quantities; for example Singapore has high GDP per capita but low fitness. On the other hand Bolivia has high fitness (world leader producer of Quinoa), but low GDP per capita. (b) The dynamical evolution of country fitness and GDP per capita of China, India and Brazil from 1992 to 2011. As time goes on, the GDP per capita of these countries increase, but the fitness of Brazil and India are constant on average while China has increased.

It is also interesting to see if there is any correlation between this new network measure (in particular country fitness) and other economic or food security indicators. A strong correlation would suggest that country fitness provides redundant information with respect to prior well-known indicators such as per-capita Gross Domestic Product (GDP/population). However, due to the high non-linearity of the map used to calculate countries fitness, we did not expect any trivial correlations to be found. Indeed, from Fig 6(a) we see that country fitness and GDP per capita are only weakly correlated (*R*^2^ = 0.194; Zero correlation test P-value < 0.001). As examples for why this is the case, Singapore, which has high GDP per capita but is an island country with low geographical and climatological diversity, consequently possesses a small basket of produced food commodities. On the other hand Bolivia has high fitness (world leader producer of Quinoa), but low GDP per capita. To further this notion that GDP and fitness are not well related, we can also investigate the dynamic relationship between country fitness and GDP per capita. Rather than a static representation, we can show country specific trajectories in the plane of country fitness and GDP per capita (three representative countries, China, Brazil and India from 1992 to 2011 shown in Fig 6(b)). It is evident that the GDP per capita of these countries increases as time goes on with country specific behavior in terms of fitness. The fitness of Brazil and India are constant on average, while China’s fitness has increased in time along with the increase in GDP per capita, suggesting an improvement in the diversification and relative production volumes (with respect to population) of food commodities. However, this latter case of China is an exception, rather than a typical behavior.

## Conclusion

In this work we have exploited country-food production data to identify subsets of countries which produce similar commodities or subsets of commodities shared by a given country allowing for visualization of correlations in large networks. We have also applied a non-linear iterative map to rank food specialization and country fitness. Our results highlight how the bipartite country-food production network is nested, reflecting that, on average, countries with high fitness producing highly specialized food commodities also produce low specialization goods, while nations with low fitness producing a small basket of food products, typically produce low specialization food commodities. These results are similar to what was found by Tacchella and collaborators for economic products [9].

We envision that the quantitative methods presented here will provide new approaches to fundamental analyses alimented by the available data of country-food security. In fact, there is increasing evidence that a comprehensive analysis of food security and sustainability, food trade must be taken in account, in order to evaluate which countries are dependent on food imports [5, 31, 32], and to estimate the impact of the intensification of the land grabbing [33]. The narrowing of diversity in crop and animal species contributing to the world’s food supplies is considered a potential threat to food security [34, 35] and should be readily identified in application of the framework presented above. For example we find that during the period 1992-2011 Ireland and New Zealand decreased their food production diversity, producing 16 and 22 different food commodities in 2011 instead of 21 and 29 in 1991, respectively resulting in a corresponding ≈ 25% decrease in fitness. We stress that this analysis can be easily applied to study production and trade networks weighted with respect to different nutritional indicators (proteins, fat, calories). As such future work can explore import and export data, food specialization with regards to secondary commodities (e.g. chocolates, breads, cheese) in order to better understand how food trade impacts each country’s fitness and specialization. There is also the ability to investigate the relation between food specialization, food price and price volatility as, combined with the presented analysis on import MSF sub-trees, it may help to develop a framework in which to detect early warning signs of local food crises.

## Supporting Information

S1 Fig The country-country similarity Minimum Spanning Forest for networks with 4 or more nodes obtained from the country-product weighted matrix of the year 2011, using the algorithm described in the Methods section of the main text. The thickness of the edges corresponds to the weight of the link (the thicker the larger).
(TIF)

S2 Fig The country-country similarity Minimum Spanning Forest for networks with 4 or more nodes obtained from the country-import weighted matrix of the year 2011, using the algorithm described in the Methods section of the main text. The thickness of the edges corresponds to the weight of the link (the thicker the larger).
(TIF)
