# Supplementary figures and images for: A Data Driven Network Approach to Rank Countries Production Diversity and Food Specialization
Source: PLoS One. 2016 Nov 10;11(11):e0165941. doi: 10.1371/journal.pone.0165941 (PMC5104443; doi:10.1371/journal.pone.0165941)

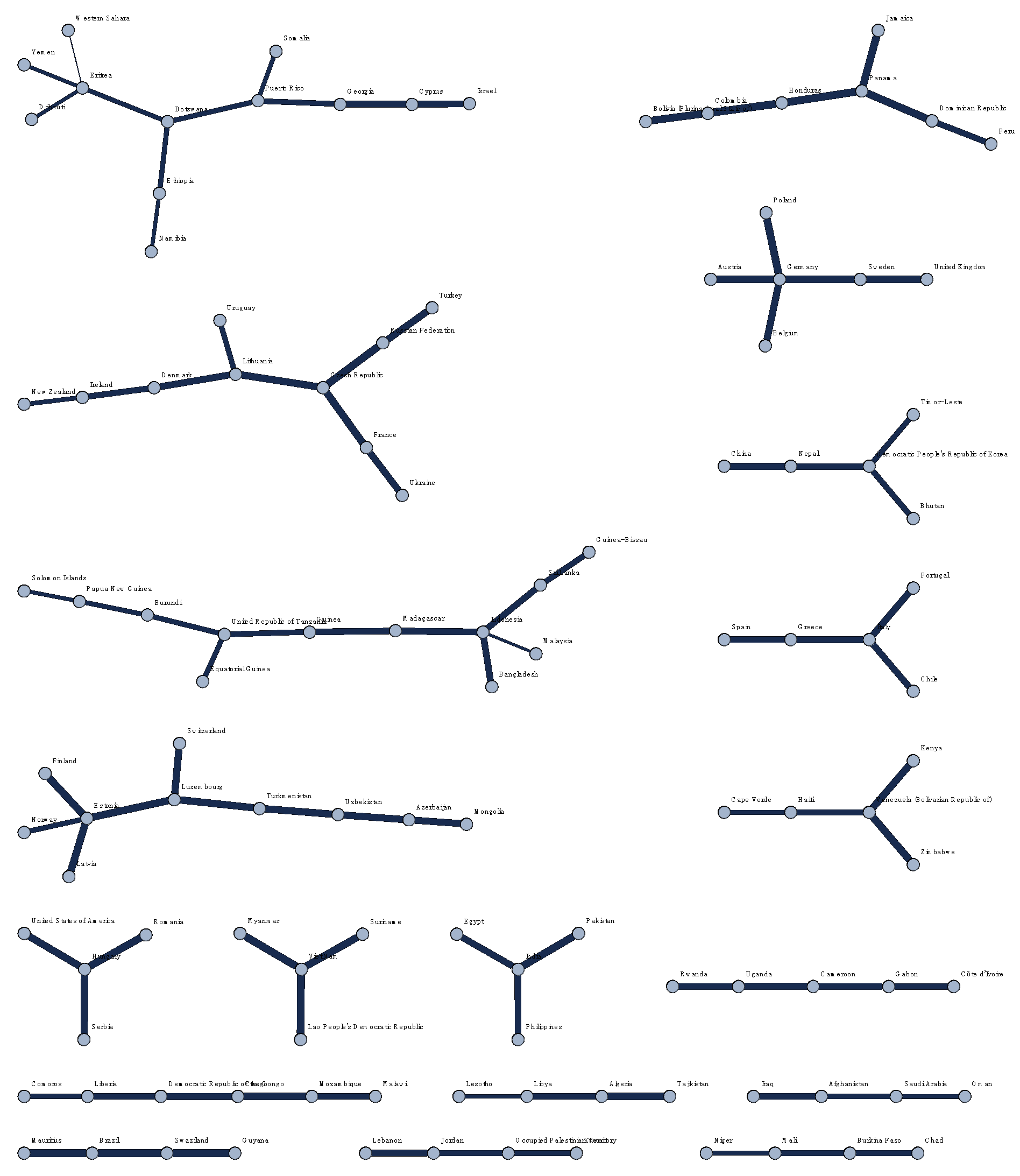

Supplement: S1 Fig — The thickness of the edges corresponds to the weight of the link (the thicker the larger). (TIF) [file pone.0165941.s001.tif]

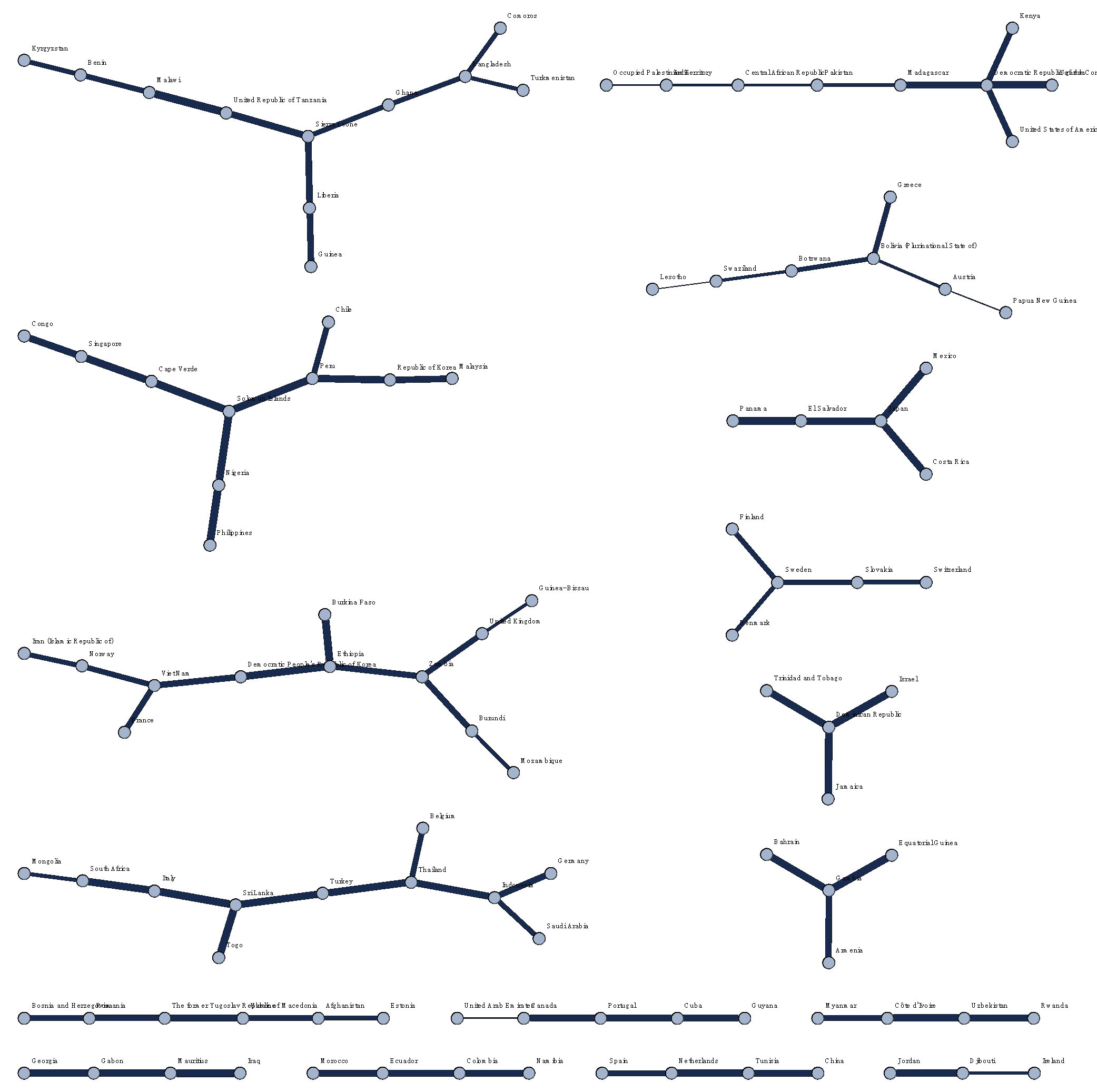

Supplement: S2 Fig — The thickness of the edges corresponds to the weight of the link (the thicker the larger). (TIF) [file pone.0165941.s002.tif]
